# Correction: Rodrigues et al. Physicochemical, Morphological, and Cytotoxic Properties of Brazilian Jackfruit (*Artocarpus heterophyllus*) Starch Scaffold Loaded with Silver Nanoparticles. *J. Funct. Biomater.* 2023, *14*, 143

**DOI:** 10.3390/jfb16120464

**Published:** 2025-12-17

**Authors:** José Filipe Bacalhau Rodrigues, Valeriano Soares Azevedo, Rebeca Peixoto Medeiros, Gislaine Bezerra de Carvalho Barreto, Maria Roberta de Oliveira Pinto, Marcus Vinicius Lia Fook, Maziar Montazerian

**Affiliations:** Academic Unit of Materials Science and Engineering, Federal University of Campina Grande, Campina Grande 58429-140, PB, Brazil

## Error in Figure

In the original publication [1], a reader had a concern regarding Figure 4. The concern was that some of the X-ray diffraction (XRD) data in Figure 4 in the ~65–80° region appeared more similar than expected. The authors re-conducted the experiment, and the updated Figure 4 appears below. 

## Text Correction

After a concern was raised about the observation that some of the X-ray diffraction (XRD) data in Figure 4 in the ~65–80° region appeared more similar than expected, the original XRD data were provided to the publisher’s office. The authors were uncertain about the source of this similarity. Therefore, the experiment was repeated to verify the results. The new XRD results, shown in this correction (Figure 4) (to replace Figure 4 in the original article), indicate no similarity in the 65–80° range of the XRD patterns. Additionally, no diffraction peaks were observed in that range, which is consistent with reports in the literature. 

After careful investigation of the new XRD results, the initial statement in the article—that the peaks “confirm” type-A crystallinity—was found to be too strong. The cited literature in the article shows characteristic diffraction peaks at 15°, 17°, 17.9°, and 22.9°. The original XRD in this research included peaks at 15.86° and 17.18°, a doublet at 22.94° and 23.88°, and additional peaks at 21.04° and 28.88°. However, the new XRD shows peaks at 15°, 17°, 17.9°, 19.7°, 23.2°, and 23.7° which are consistent with type-A crystalline structures and align closely with the data reported in the literature. The repeated XRD therefore provides better evidence and a clearer description of the XRD patterns. 

It was also noted that the original figure description contained errors in curve labeling. In addition, the initial statement that the scaffold peaks were “identical” to those of the starch diffractogram was inaccurate. While the scaffold and starch curves share major peaks, they are not strictly identical. This description has been revised to state that the peaks are “similar” but not identical.

Furthermore, it should be noted that the repeated XRD analysis was performed on a previously stored sample (kept for approximately three years in sealed containers at ambient temperature and humidity), as the originally tested material was no longer available. We acknowledge that repeating experiments on stored samples is not an ideal practice and may have contributed to the partial loss of crystallographic features observed. Factors such as storage conditions, environmental humidity, or natural sample aging could explain the reduced crystallographic manifestation. Nevertheless, the peaks that remain are consistent with type-A crystalline structures, and this partial loss does not compromise the validity of the overall conclusions.

The description of Figure 4 in Section 3.3 has been updated as follows:

Figure 4 depicts the XRD pattern of the jackfruit starch, starch scaffold, and starch–AgNPs scaffold. Jackfruit starch’s XRD in Figure 4A shows diffraction peaks at 15°, 17°, 17.9°, 19.7°, 23.2°, and 23.7°, which are consistent with type-A crystalline structures and align closely with the data reported in the literature [72–74].

Three peaks (17°, 19.2°, and 23.6°) observed in the starch scaffold (Figure 4B) appeared at positions similar to those in the starch diffractogram (Figure 4A), but with amorphous characteristics and reduced intensity, indicating a partial loss of the type-A crystalline structure of jackfruit starch after processing (crystallinity = 16.95% by Rietveld refinement). Studies indicate that plasticized starch tends to form a V-type crystalline structure, which was indicated by the appearance of a slight shoulder around 17° and 19.2° (Figure 4B) [75]. In manufacturing starch scaffolds, glycerol was used, which acts as a plasticizer [76]. Therefore, it is suggested that, during processing, there was a change in the crystalline structure of starch through its interaction with glycerol.

The authors state that the scientific conclusions are unaffected. This correction was approved by the Academic Editor. The original publication has also been updated.

## Figures and Tables

**Figure 4:**
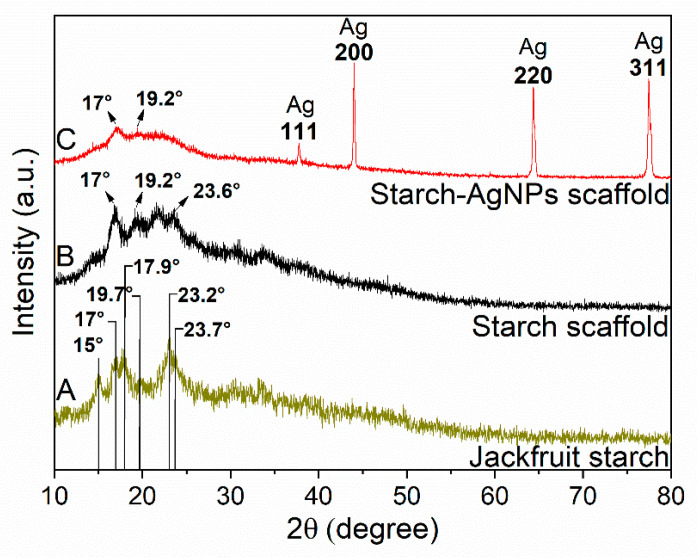
XRD patterns of the (**A**) jackfruit starch, (**B**) starch scaffold, and (**C**) starch–AgNPs scaffold.
